# Next-generation sequencing facilitates detection of the classic E13-A20 EML4-ALK fusion in an ALK-FISH/IHC inconclusive biopsy of a stage IV lung cancer patient: a case report

**DOI:** 10.1186/s13000-016-0581-4

**Published:** 2016-11-18

**Authors:** Anna-Lena Volckmar, Volker Endris, Farastuk Bozorgmehr, Clemens Lier, Carlota Porcel, Martina Kirchner, Jonas Leichsenring, Roland Penzel, Michael Thomas, Peter Schirmacher, Arne Warth, Albrecht Stenzinger

**Affiliations:** 1 Institute of Pathology, University Hospital Heidelberg, Im Neuenheimer Feld 224, 69120 Heidelberg, Germany; 2 Thoracic Oncology, Thoraxklinik, University of Heidelberg, and Translational Lung Research Center Heidelberg, 69120 Heidelberg, Germany; 3 German Center for Lung Research (DZL), Heidelberg, Germany; 4 German Cancer Consortium (DKTK), Heidelberg, Germany

**Keywords:** ALK, FISH, NGS, immunohistochemistry, Variant 1, EML4-ALK

## Abstract

**Background:**

Inhibition of the oncogenic fusion-gene EML4-ALK is a current first-line approach for patients with stage IV non-small cell lung cancer. While FISH was established as the gold standard for identifying these patients, there is accumulating evidence that other methods of detection, i.e., immunohistochemistry and next-generation sequencing (NGS), exist that may be equally successful. However, the concordance of these methods is under investigation.

**Case presentation:**

Adding to the current literature, we here report a 56 year old female never-smoker with stage IV lung adenocarcinoma whose biopsy was IHC and FISH inconclusive but positive in NGS. Retroactive profiling of the resection specimen corroborated fusion reads obtained by NGS, FISH-positivity and showed weak ALK-positivity by IHC. Consequently, we diagnosed the case as ALK-positive rendering the patient eligible to crizotinib treatment.

**Conclusions:**

With IHC on biopsy material only, this case would have been overlooked withholding effective therapy.

## Background

Genetic profiling of stage IV lung adenocarcinomas is state of the art to identify patients who are eligible to tyrosine kinase inhibitors targeting EGFR, and fused genes involving *ALK*, *ROS* and *RET*. Following the discovery of the oncogenic *EML4-ALK* fusion gene by Soda et al. [1], a series of trial data (phase I-III) published between 2010 and 2014 demonstrated the efficacy of crizotinib [2–4], a tyrosine kinase inhibitor that was originally developed to target MET [5] in non-small-cell lung cancer patients and led to rapid approval of the FDA and subsequently the EMA. The PROFILE 1014 study established crizotinib as a first-line therapy yielding a better progression-free survival than chemotherapy regimens used at that time [4]. The phase I trial by Kwak et al. [2] used fluorescence in-situ hybridization (FISH) employing a cut-off of > 15% tumor cells harboring split signals to determine *ALK*-positive tumors. A retrospective analysis of a subset of this cohort showed orthogonal methods (i.e., RT-PCR and IHC) to correlate with FISH data to some extent. Consequently, the FDA approved method for the detection of *ALK* rearrangements is FISH while the EMA allows use of any method in Europe that correctly identifies patients whose tumor harbors ALK fusions. Over the last 5 years there is accumulating data that particularly immunohistochemical (IHC) detection of ALK may be a suitable surrogate marker for rearrangement events involving ALK [6–9]. However, while concordance between IHC and FISH is high [9, 10], discordant cases exist and a number of studies reported ALK- IHC-positive cases that are negative by FISH [11, 12].

## Patient and methods

Written informed consent was obtained, and the case report follows the CARE guidelines [13]. We performed ALK immunohistochemistry using the D5F3 clone on a BenchMark XT autostainer with the UltraView DAB detection kit. The antibody is described to be sensitive to all EM4-ALK variants [6, 10, 14, 15]. FISH analysis and next generation sequencing were carried out as described previously [16]. Briefly, we employed the ALK dual color break apart probe (Zytovision, Germany) that detects rearrangements involving the chromosomal region 2p23.1–p23.2. To this end, the probe labeled with the green fluorochrome hybridizes proximal to the ALK gene breakpoint region at 2p23.1–p23.2 and the probe with the orange fluorochrome hybridizes distal to the ALK gene breakpoint region at 2p23.2. A normal interphase cell without translocation of this locus shows yellow fusions signals whereas separation of signals (a split signal) indicates translocation/inversion.

## Case presentation

**Fig. 1:**
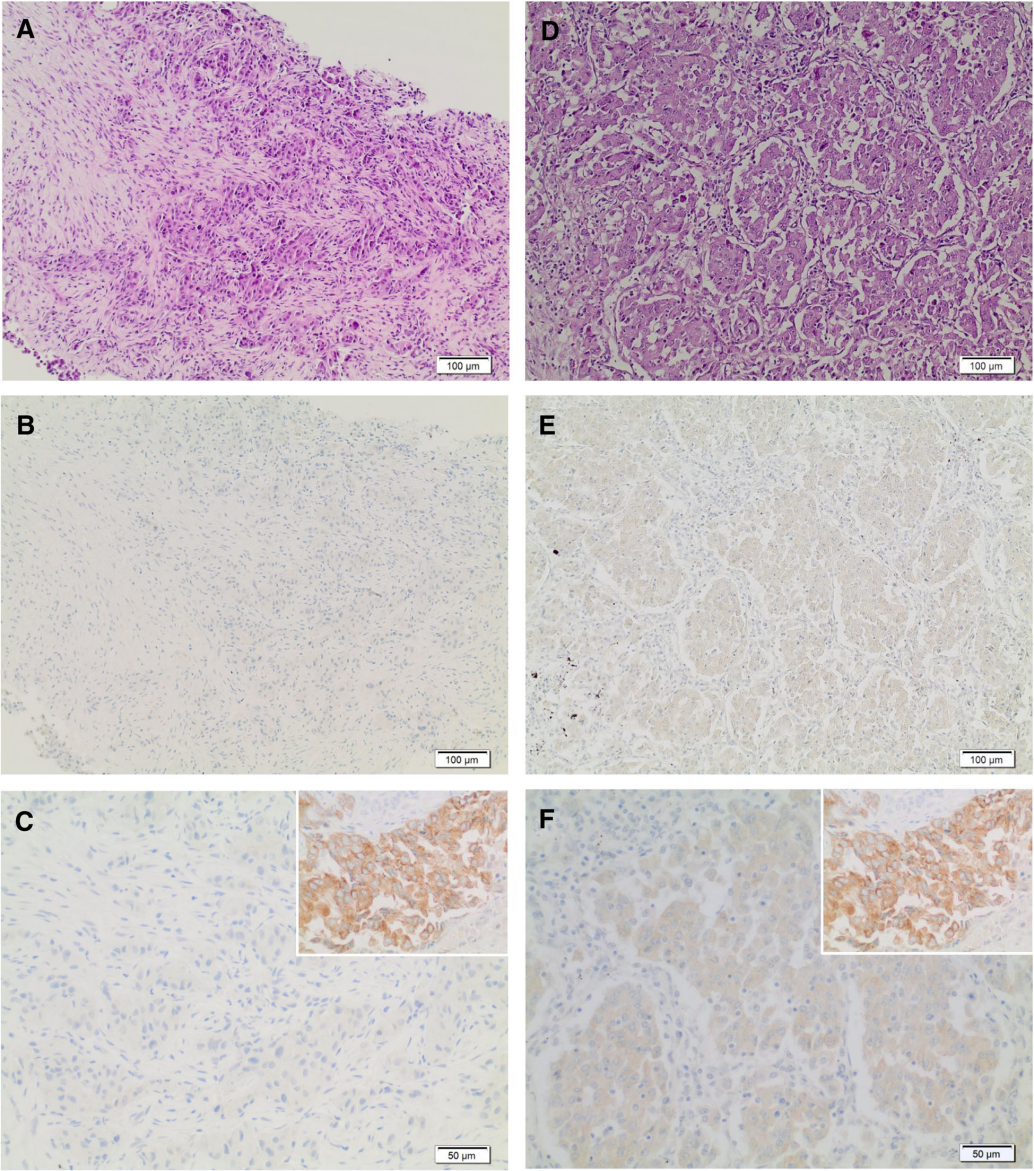
HE-staining and ALK-IHC of the NSCLC (scale bar indicates magnification). **a** HE-stained biopsy specimen, **b** Biopsy specimen: Overview of ALK-IHC staining, **c** Biopsy specimen: High power view of ALK-IHC staining. Insert shows a positive case with strong staining serving as internal control., **d** HE-stained surgical specimen, **e** Surgical specimen: Overview of ALK-IHC staining, **f** Surgical specimen: High power view of ALK-IHC staining. Insert shows a positive case with strong staining serving as internal control

**Fig. 2:**
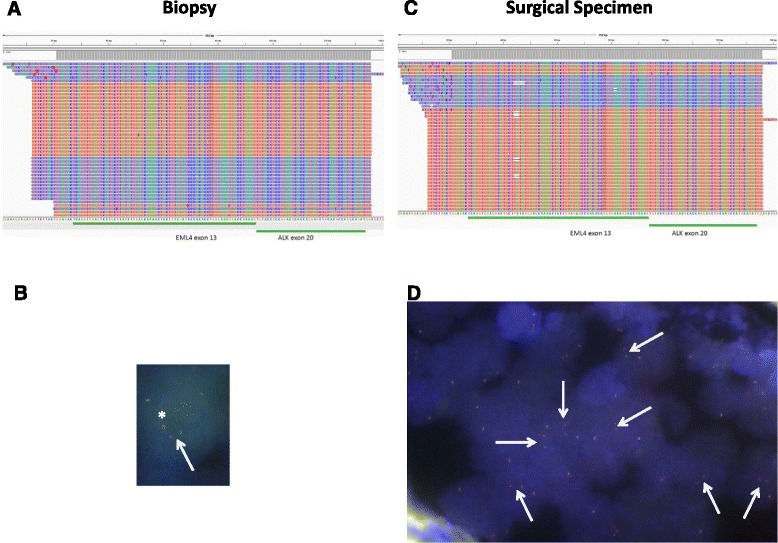
Genetic analysis of the biopsy and surgical specimen. **a** Reads of the E13-A20 EML4-ALK fusion transcript in the biopsy specimen. **b** Reads of the E13-A20 EML4-ALK fusion transcript in the surgical specimen. **c** FISH analysis of the biopsy specimen. Asterisk marks fused signal, arrow points to splits signal. **d** FISH analysis of the surgical specimen. Arrows indicate split signals. Both FISH pictures were taken at 100× magnification using oil

We here report the case of a 56 year old Caucasian female never-smoker, who was initially diagnosed with clinical stage IIIA adenocarcinoma of the lung in 2014. The patient underwent surgical resection of the primary and adjuvant therapy (4 cycles of adjuvant chemotherapy with cisplatin/vinorelbine). Two years after the resection of the tumor, the patient developed distant metastatic disease (Fig. 1a) and was eventually biopsied at the right iliac bone for pathological evaluation and genetic testing of druggable targets. We employed combined testing for fusions and mutations as described previously [16], and detected fusion of EML4 exon 13 with ALK exon 20, which is one of the most common fusion variants in lung adenocarcinomas (variant 1) [17]. Consistent with this result, we neither detected mutated *EGFR* or *KRAS*, or fusions involving *ROS* or *RET*. In parallel, IHC staining for ALK was applied. While massive parallel sequencing showed 4163 fusion reads indicating expression of the fusion gene (Fig. 2a) IHC staining was largely negative except for very few intermingled cells that may show exceedingly faint ALK expression at high magnification, which cannot be reliably subtracted from background signal in routine diagnostics (Fig. 1b, c). Repeated IHC staining for ALK yielded similar results (data not shown) and orthogonal FISH analysis showed impaired hybridization with only very few cells exhibiting probe signals including splits at low intensity (Fig. 2b). As the current guidelines require that at least 15% of the tumor cells show split (or single red) signals [18, 19], FISH data of the biopsy specimen were inconclusive and thus non-informative. HE-stained slides suggested crush and thermal artifacts that are known to influence IHC performance. These divergent findings prompted us to perform additional molecular profiling of the primary tumor (Fig. 1d) that was resected two years earlier. In keeping with the results observed in the biopsy specimen, we detected the very same gene fusion at even higher read counts (9639 fusion reads; Fig. 2c). In accord with these genetic data, we detected clear-cut split signals in all cancer cells by FISH (Fig. 2d). Corresponding IHC staining of ALK showed weak ALK expression (Fig. 1e, f). Based on the integrated view of these results, we designated this case as positive for a fusion event involving ALK thus rendering the patient eligible to treatment with crizotinib.

## Conclusion

The major trials that lead to the approval of crizotinib for the treatment of EML4-ALK positive non-small cell lung cancer patients employed genetic testing by FISH to identify responders. Consequently, the guidelines for molecular testing of NSCLC issued by the College of American Pathologists (CAP), International Association for the Study of Lung Cancer (ISLAC), and Association for Molecular Pathology (AMP) in 2013 [19], recommend dual color FISH for the detection of fusion events that predict response towards therapy, and IHC as a putative screening method for cases that require FISH testing. Over the last 3 years, a number of studies have reported a high concordance of FISH and IHC data. However, concordance results did not reach 100% and a large study of 3244 cases identified a substantial number of cases that were either FISH-positive/IHC-negative or FISH-negative/IHC-positive [20]. The authors of this study conclude that using either test alone would have failed to detect about 25% of ALK-positive cases. A recent study by Pekar-Zlotin et al. [21] concluded that FISH-based testing may miss a significant number of patients who are eligible to ALK inhibition and observed a sensitivity and specificity of 42.9% and 97.7% for FISH and 100 and 97.7% for IHC compared to an NGS-based approach that served as a gold standard for ambiguous cases. Based on their experience with 51 patients they suggested that NGS should be applied in cases with inconclusive IHC staining. Costs for an NGS-based approach are currently higher than for a single FISH analysis if one compares both methodologies on the basis of a single analysis (e.g., ALK testing). However, the NGS methodology allows simultaneous upfront detection of clinically relevant point mutations, amplifications, deletions and gene fusions in a one-stop shop which is highly efficient and tissue sparing [16]. The latter is a crucial point when it comes to small biopsies where tumor cellularity may be low. NGS also provides data on the fusion partners which may become relevant in the future. With dropping costs, advantageous NGS-based assays will most likely replace combinatory approaches of e.g., Sanger sequencing (for detection of mutant EGFR) and FISH analysis, which are still the mainstay of molecular diagnostics and which are also used by us as robust and orthogonal methods.

Here, we show a case where ALK-IHC was negative in the biopsy material, except for very few intermingled cells exhibiting extremely faint positivity that cannot be reliably discriminated from background signals. Similarly, FISH showed poor hybridization performance and results were non-informative. Concomitant targeted deep sequencing, however, that was implemented at our institution [16], showed the classic variant 1 fusion between EML4 and ALK affecting exons 13 and exon 20, respectively. These divergent results prompted us to profile the surgical resection specimen by NGS where we observed the very same fusion event and, in accord with this finding, detected clear-cut split signals in the FISH analysis as well as weak but positive ALK immunostaining. Weak IHC signals in FISH-positive ALK cases are well known and have been reported in the literature [22–24]. While the underlying cause is unclear, antibody avidity may account for some of the cases. As all formalin and paraffin embedded specimens at our institution are processed according to standard protocols and standard operating procedures that are continuously monitored by quality management, a systematic error is unlikely. We did not observe any evidence for genetic heterogeneity in the surgical specimen, i.e., the ALK fusion event was present in all tumor cells which is consistent with the concept of mutual exclusivity with other main drivers, e.g., mutant EGFR, and the notion that ALK fusion are truncal mutations. As hybridization performance for FISH was poor in the biopsy specimen and targeted RNA sequencing does not provide data on a single cell level, we cannot exclude genetic heterogeneity in the biopsy but it appears unlikely for the reasons mentioned above. In theory, epigenetic silencing could be causal for the absent protein expression in the biopsy specimen. However, our targeted RNA-seq data clearly demonstrate transcription of the fusion sequence (i.e. no gene silencing) and is also in line with the notion that downregulation of the main driver would be disadvantageous for tumor growth and spread. In summary, taken the data of both specimens into account, we diagnosed an ALK-positive lung adenocarcinoma rendering the patient eligible to crizotinib.

While we cannot fully explain the negative FISH and IHC results on the biopsy specimen, conventional morphology of the small iliac biopsy suggests that crush or thermal artifacts introduced by cauterization and surgical instruments likely account for the drop out. As our quality controlled NGS approach yielded clear results for both the biopsy as well as for the surgical specimen, this case illustrates that NGS-based detection of ALK fusions is particularly advisable in cases where FISH and IHC results are inconclusive or tissue artifacts are suspected to avoid false negative results that may withhold effective therapy. This being said, further studies are warranted to comprehensively investigate the diagnostic utility and power of the three different methods in specific clinical settings.

## Abbreviations

EMA: European Medicines Agency
FDA: US Food and Drug Administration
FISH: Fluorescence In-Situ Hybridization
HE: Hematoxylin and Eosin
NGS: Next Generation Sequencing

## References

[1] Soda M, Choi YL, Enomoto M, Takada S, Yamashita Y, Ishikawa S (2007). Identification of the transforming EML4-ALK fusion gene in non-small-cell lung cancer. Nature.

[2] Kwak EL, Bang Y-J, Camidge DR, Shaw AT, Solomon B, Maki RG (2010). Anaplastic lymphoma kinase inhibition in non-small-cell lung cancer. N Engl J Med.

[3] Shaw AT, Kim D-W, Nakagawa K, Seto T, Crino L, Ahn M-J (2013). Crizotinib versus chemotherapy in advanced ALK-positive lung cancer. N Engl J Med.

[4] Solomon BJ, Mok T, Kim D-W, Wu Y-L, Nakagawa K, Mekhail T (2014). First-line crizotinib versus chemotherapy in ALK-positive lung cancer. N Engl J Med.

[5] Christensen JG, Zou HY, Arango ME, Li Q, Lee JH, McDonnell SR (2007). Cytoreductive antitumor activity of PF-2341066, a novel inhibitor of anaplastic lymphoma kinase and c-Met, in experimental models of anaplastic large-cell lymphoma. Mol Cancer Ther.

[6] Conklin CMJ, Craddock KJ, Have C, Laskin J, Couture C, Ionescu DN (2013). Immunohistochemistry is a reliable screening tool for identification of ALK rearrangement in non-small-cell lung carcinoma and is antibody dependent. J Thorac Oncol.

[7] Paik JH, Choe G, Kim H, Choe J-Y, Lee HJ, Lee C-T (2011). Screening of anaplastic lymphoma kinase rearrangement by immunohistochemistry in non-small cell lung cancer: correlation with fluorescence in situ hybridization. J Thorac Oncol.

[8] Park HS, Lee JK, Kim D-W, Kulig K, Kim TM, Lee S-H (2012). Immunohistochemical screening for anaplastic lymphoma kinase (ALK) rearrangement in advanced non-small cell lung cancer patients. Lung Cancer.

[9] McLeer-Florin A, Moro-Sibilot D, Melis A, Salameire D, Lefebvre C, Ceccaldi F (2012). Dual IHC and FISH testing for ALK gene rearrangement in lung adenocarcinomas in a routine practice: a French study. J Thorac Oncol.

[10] Wynes MW, Sholl LM, Dietel M, Schuuring E, Tsao MS, Yatabe Y (2014). An international interpretation study using the ALK IHC antibody D5F3 and a sensitive detection kit demonstrates high concordance between ALK IHC and ALK FISH and between evaluators. J Thorac Oncol.

[11] Rosoux A, Pauwels P, Duplaquet F, D’Haene N, Weynand B, Delos M (2016). Effectiveness of crizotinib in a patient with ALK IHC-positive/FISH-negative metastatic lung adenocarcinoma. Lung Cancer.

[12] Peled N, Palmer G, Hirsch FR, Wynes MW, Ilouze M, Varella-Garcia M (2012). Next-generation sequencing identifies and immunohistochemistry confirms a novel crizotinib-sensitive ALK rearrangement in a patient with metastatic non-small-cell lung cancer. J Thorac Oncol.

[13] Gagnier JJ, Kienle G, Altman DG, Moher D, Sox H, Riley D. The CARE guidelines: consensus-based clinical case reporting guideline development. BMJ Case Rep. 2013;2013. doi:10.1136/bcr-2013-201554. 10.1186/1752-1947-7-223 PMC3844611 24228906

[14] Taheri D, Zahavi DJ, Del Carmen Rodriguez M, Meliti A, Rezaee N, Yonescu R (2016). For staining of ALK protein, the novel D5F3 antibody demonstrates superior overall performance in terms of intensity and extent of staining in comparison to the currently used ALK1 antibody. Virchows Arch.

[15] Mino-Kenudson M, Chirieac LR, Law K, Hornick JL, Lindeman N, Mark EJ (2010). A novel, highly sensitive antibody allows for the routine detection of ALK-rearranged lung adenocarcinomas by standard immunohistochemistry. Clin Cancer Res.

[16] Pfarr N, Stenzinger A, Penzel R, Warth A, Dienemann H, Schirmacher P (2016). High-throughput diagnostic profiling of clinically actionable gene fusions in lung cancer. Genes Chromosomes Cancer.

[17] Cancer Genome Atlas Research Network. Comprehensive molecular profiling of lung adenocarcinoma. Nature. 2014;511:543–50. 10.1038/nature13385 PMC4231481 25079552

[18] von Laffert M, Stenzinger A, Hummel M, Weichert W, Lenze D, Warth A (2015). ALK-FISH borderline cases in non-small cell lung cancer: Implications for diagnostics and clinical decision making. Lung Cancer.

[19] Lindeman NI, Cagle PT, Beasley MB, Chitale DA, Dacic S, Giaccone G (2013). Molecular testing guideline for selection of lung cancer patients for EGFR and ALK tyrosine kinase inhibitors: guideline from the College of American Pathologists, International Association for the Study of Lung Cancer, and Association for Molecular Pathology. J Thorac Oncol.

[20] Cabillic F, Gros A, Dugay F, Begueret H, Mesturoux L, Chiforeanu DC (2014). Parallel FISH and immunohistochemical studies of ALK status in 3244 non-small-cell lung cancers reveal major discordances. J Thorac Oncol.

[21] Pekar-Zlotin M, Hirsch FR, Soussan-Gutman L, Ilouze M, Dvir A, Boyle T (2015). Fluorescence in situ hybridization, immunohistochemistry, and next-generation sequencing for detection of EML4-ALK rearrangement in lung cancer. Oncologist.

[22] Conde E, Suarez-Gauthier A, Benito A, Garrido P, Garcia-Campelo R, Biscuola M (2014). Accurate identification of ALK positive lung carcinoma patients: novel FDA-cleared automated fluorescence in situ hybridization scanning system and ultrasensitive immunohistochemistry. PLoS One.

[23] Sholl LM, Weremowicz S, Gray SW, Wong K-K, Chirieac LR, Lindeman NI, Hornick JL (2013). Combined use of ALK immunohistochemistry and FISH for optimal detection of ALK-rearranged lung adenocarcinomas. J Thorac Oncol.

[24] von Laffert M, Warth A, Penzel R, Schirmacher P, Kerr KM, Elmberger G (2014). Multicenter immunohistochemical ALK-testing of non-small-cell lung cancer shows high concordance after harmonization of techniques and interpretation criteria. J Thorac Oncol.

